# *De Novo* Transcriptome and Small RNA Analysis of Two Chinese Willow Cultivars Reveals Stress Response Genes in *Salix matsudana*

**DOI:** 10.1371/journal.pone.0109122

**Published:** 2014-10-02

**Authors:** Guodong Rao, Jinkai Sui, Yanfei Zeng, Caiyun He, Aiguo Duan, Jianguo Zhang

**Affiliations:** 1 State Key Laboratory of Tree Genetics and Breeding, Research Institute of Forestry, Chinese Academy of Forestry, Beijing, Republic of China; 2 Key Laboratory of Tree Breeding and Cultivation, State Forestry Administration, Research Institute of Forestry, Chinese Academy of Forestry, Beijing, Republic of China; George Washington University School of Medicine and Health Sciences, United States of America

## Abstract

*Salix matsudana* Koidz. is a deciduous, rapidly growing, and drought resistant tree and is one of the most widely distributed and commonly cultivated willow species in China. Currently little transcriptomic and small RNAomic data are available to reveal the genes involve in the stress resistant in *S. matsudana*. Here, we report the RNA-seq analysis results of both transcriptome and small RNAome data using Illumina deep sequencing of shoot tips from two willow variants(*Salix. matsudana* and *Salix matsudana* Koidz. cultivar ‘Tortuosa’). *De novo* gene assembly was used to generate the consensus transcriptome and small RNAome, which contained 106,403 unique transcripts with an average length of 944 bp and a total length of 100.45 MB, and 166 known miRNAs representing 35 miRNA families. Comparison of transcriptomes and small RNAomes combined with quantitative real-time PCR from the two *Salix* libraries revealed a total of 292 different expressed genes(DEGs) and 36 different expressed miRNAs (DEMs). Among the DEGs and DEMs, 196 genes and 24 miRNAs were up regulated, 96 genes and 12 miRNA were down regulated in *S. matsudana*. Functional analysis of DEGs and miRNA targets showed that many genes were involved in stress resistance in *S. matsudana*. Our global gene expression profiling presents a comprehensive view of the transcriptome and small RNAome which provide valuable information and sequence resources for uncovering the stress response genes in *S. matsudana*. Moreover the transcriptome and small RNAome data provide a basis for future study of genetic resistance in *Salix*.

## Introduction

The increasing concern about climate change and energy security has resulted in the focusing on the economic importance of *Salix* species, given their utility for bioenergy production. However, plants have to tolerate many abiotic and biotic stresses, which are serious threats to agriculture and forestry. These common environmental stresses include salinity, extreme temperatures, drought, chemical toxicity, oxidative stress, pests, and pathogen infection. To exploit their potential for renewable energy, willows need to be kept free of pests and diseases and the yield should be improved without significantly increasing the requirement for fertilizers and water [1].

Previous studies have reveled many *Salix* species have the capacity to tolerate abiotic and biotic stress. The willow species *S. caprea* and *S. cinera* are grown on nutrient- deficient and industrially- contaminated soils; thus, they sever as biological filters for waste water and sludge disposal [2]. Nine different clones of six species of *Salix* (*Salix cordata* Muhlenb. non Michaux, *S*. *fragilis* L., *S. caprea* L., *S. cinerea* L., *S. burjatica* Nazarov. and *S. viminalis* L.) and one hybrid (*S.* x *calodendron* Wimm.) were exposed to heavy metals in solution culture showed reduced phytotoxicity and increased metal resistance [2]. Two Russian willow species, *Salix viminalis* and *Salix dasyclados* have shown strong genetic control of freezing resistance at during autumn [3]. *Salix caprea*, *Salix repens* and their F1 hybrids had the ability to tolerate different insects and pathogens [4].

Many willow species are characterized by their biological property and ability to adapt to stressful conditions, including high biomass, tolerance to heavy metals, rapid growth, and tolerance of flooding [5]. *Salix matsudana* Koidz. is a deciduous, rapidly growing tree that is native to northeastern China, and is one of the most widely distributed and commonly cultivated willow species in China [6]. The Chinese name for *S. matsudana* is ‘Han Liu’, where ‘Liu’ means willow and ‘Han’ means that the willow is drought tolerant. *Salix matsudana* Koidz. cultivar ‘Tortuosa’ is widely propagated and planted as an ornamental tree in China because of its attractive twisted stem. This variant is also a water loving plant, which is often cultivated near the river.

The high-throughput next generation sequencing technology such as RNA-seq and Digital Gene Expression (DGE) is an efficient method to discover genes in an unbiased manner [7]–[10]. Measurement of mRNA and miRNA expression levels, and elucidation of the regulatory relationship between miRNA and their corresponding mRNA targets are crucial for understanding many pathways and biological systems including plant development, and biotic and abiotic stress responses [11].

However, there have been a few studies probing the ability of *S. matsudana* withstand drought stress. In this study, we performed a transcriptome and miRNA sequencing from one-year-old stem shoots of *Salix matsudana* Koidz. and *Salix matsudana* Koidz. cultivar ‘Tortuosa’ using deep sequencing technology to discover the differentially expressed genes and miRNAs in stem shoots of the two varieties of *Salix*. Our results showed that 196 transcripts, which are mainly related to plant abiotic and biotic stress responses, are commonly up-regulated in *S. matsudana*. These up-regulated genes in *S. matsudana* included a set of biotic and abiotic stress response genes, such as pathogen defense, insect resistance, antibiotics tolerance, antioxidant, and hormone related genes. Functional analysis of miRNA targets also showed that many genes were involved in stress resistance in *S. matsudana*. The transcriptome and small RNAome reported here on the two Chinese willow cultivars significantly enhance our knowledge of stress response genes in *Salix*.

## Methods

### Plant materials

Shoots from a natural population of *Salix matsudana* and *Salix matsudana* Koidz. cultivar ‘Tortuosa’ were harvested in spring 2013 at Beijing Botanical Garden of China. To ensure that there absolute minimal differences in the growth conditions, all shoots were sampled within the distribution of 30×30 m^2^. Samples were frozen in liquid nitrogen immediately for the extraction of total RNA.

This study was carried out in strict accordance with the recommendations in the Guide for Observational and field studies (http://www.plosone.org/static/publication). All necessary permits were obtained for the described field studies. The sampling of all individuals of *Salix. matsudana* and *Salix matsudana* Koidz. cultivar ‘Tortuosa’ were approved by Xiping Zheng, director of Beijing Botanical Garden of China.

### Libraries construction and deep sequencing

For each group of samples, equal numbers of shoots from five individual plants were pooled for the total RNA extraction with the TRIZOL reagent according to the manufacturer' instructions (Invitriogen). Small RNA and transcriptome sequencing, used the total RNA from the different pooled samples. After eliminating traces of genomic DNA by treatment with DNase I, an automated capillary gel electrophoresis was used to assess RNA quality and quantity using a Bioanalyzer 2100 with RNA 6000 Nano Labchips (Agilent Technologies Ireland, Dublin, Ireland). sRNA and Transcriptome sequencing libraries were prepared using an Illumina Small RNA Sample Prep Kitand an Illumina TruSeq RNA Sample Prep Kit, respectively. After the two libraries had been prepared, raw reads generated by using the Illumina Hiseq 2000 were initially processed to get clean reads. Then, all the clean reads were assembled using a de novo assembly program Trinity [12], [13].

### Gene validation and quantitative real-time PCR analysis

Ten selected genes specific PCR primers (Table S2) were designed corresponding to the conserved region of willow cDNA sequences from the sequenced database. PCR was performed in a total volume of 50 µl containing 2.5 mmol/L Mg^2+^, 0.15 mmol/L dNTPs, 0.4 mmol/L of each primer, 15 ng cDNA and 0.5 U PFU DNA polymerase (NEB). The PCR products were purified and ligated into the PMD19-T vector (Takara Bio), and then transformed into *E. coli* Jm109. After sequencing the positive clones with ABI 3730 (Applied Biosystems, USA), the sequences were validated to the corresponding transcriptome seqences.

Quantitative real-time PCR of mature miRNA was performed following the high-stringency protocol in which a poly A polymerase was used to add a poly A tail. The selected genes real-time quantitative PCR were conducted using the SYBR Green Perfect (Takara) and StepOnePlus™ System (Applied Biosystems). All of the PCR products were sequenced and the dissociation curve was analyzed to verify the amplification specificity. The purified PCR products were used to make standard curve for establishing a quantitative correlation between the CT values and the transcript copy numbers [14]. Each qRT-PCR reaction was repeated at least three times, and each standard curve contains at least 5 points. 5.8 s rRNA and *Populus* actin gene were used for reference gene for miRNA and transcripome genes expression validation, respectively.

### Small RNA prediction and identification of new mi RNAs in willow

Small interfering RNA (siRNA) has their own structure feature, which is a 20–25 nt long double-stranded RNA, and each strand of which is 2 nt longer than the other at the 3′ end. According to this, reads were aligned with each other in order to find potential siRNA candidate by using the alignment software Bowtie combined with Perl scripts. To identify new miRNA, sRNAs with non-annotation information were subjected to a secondary structure prediction by using the software RNAfold [15]. Further identification of new miRNA was conducted by Mireap (http://sourceforge.net/projects/mireap/).

## Result

### Transcriptome sequencing by RNA-seq and *de novo* assembly

**Figure 1:**
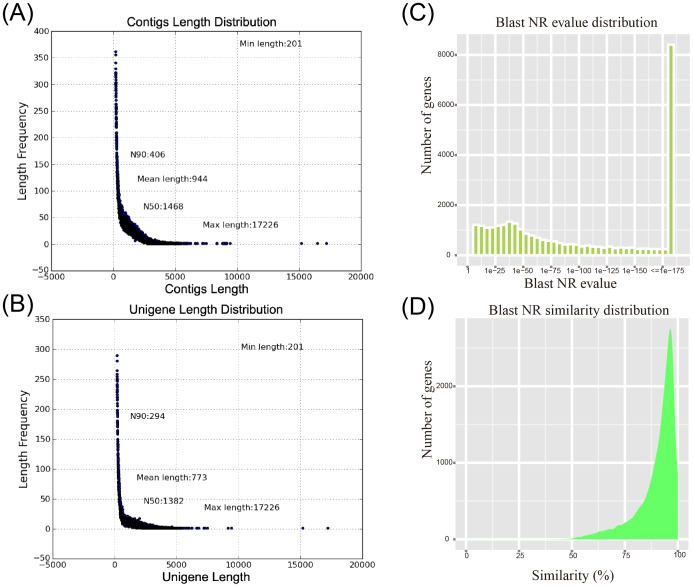
The length distribution of contigs and unigenes, and E-value distribution and similarity distribution of Blast NR hits. Minimum length, N90; mean length, N50; and maximum length of contigs and unigenes are shown in their corresponding positions. (**A**) Contigs length distribution. (**B**) Unigene length distribution. (**C**) E-value distribution of Blast NR hits for matched unigene sequences. (**D**) Similarity distribution of Blast NR hits for each unigene.

Two RNA-seq libraries were prepared from the total RNA extracted from *Salix matsudana* (‘SM’) and *Salix matsudana* Koidz. cultivar ‘Tortuosa’ (‘SMT’) and were subjected to pair-end read (PE) sequencing using Illumina platform. We removed reads with adaptors, reads with unknown nucleotides larger than 5%, and low quality reads to obtain clean PE reads in SM and SMT samples. A total of 60,521,772 clean PE reads consisting of 6,506,875,453 nucleotides (nt) with an average GC content of 45.11% were obtained in the SM sample while 58,648,772 clean PE reads consisting of 6,292,916,298 nt with an average GC content of 45.14% were obtained in the SMT sample (Table 1). An overview of the raw reads data is given in Figure S1. All high-quality clean reads were assembled into 106,430 contigs with an average length of 944 bp having a minimum length of 201 bp and a maximum length of 17,226 bp (Figure 1A). The contigs were further joined into 48,817 unigenes with an average length of 773 bp, a N50 length of 1,468 bp and a N90 length of 294 bp (Figure 1B).

**Table 1:** Statistics of transcriptome output sequencing.

Samples	SM	SMT
Total raw reads	61,958,422	60,131,098
Total clean reads	60,521,772	58,648,772
Total clean nucleotides (nt)	6,506,875,453	6,292,916,298
Q20 percentage	97.55%	98.14%
N percentage	0.06%	0.10%
GC percentage	45.11%	45.14%

### Functional annotation and classification of the assembled unigenes

Gene annotation was performed based on sequence homologies to the databases of NCBI non-redundant protein (Nr), NCBI nucleotide sequence (Nt), Kyoto Encyclopedia of Genes and Genomes (KEGG), Swiss-Prot protein, Protein family, Gene Ontology (GO), and Cluster of Orthologous Groups (COGs) using BLAST2GO. In total, 2,592 (5.2% of all unigenes) unigenes annotated in all databases, and 34,049 (69.74% of all unigenes) significantly matched a sequence in at least one of the public databases (Table 2). For the nr annotations, 29,657 of unigenes (60.75%) were found to have matches in the database. Further analysis of the BLAST data showed that 64.11% of the top hits had strong homology with the E-value <1.0e^−50^ while 35.89% of the matched sequences showed moderate homology with the E-value between 1.0e^−5^ and 1.0e^−50^ (Figure 1C).

**Table 2:** statistics of functional annotation of unigenes in public databases.

Public database	Number of Unigenes	Percentage (%)
Annotated in NR	29,657	60.75
Annotated in NT	30,551	62.58
Annotated in KEGG	5,444	11.15
Annotated in SwissProt	19,354	39.64
Annotated in PFAM	17,869	36.6
Annotated in GO	23,249	47.62
Annotated in KOG/COG	10,073	20.63
Annotated in all Databases	2,592	5.3
Annotated in at least one Database	34,049	69.74
Total Unigenes	48,817	100

The identity distribution pattern showed that 79.44% of the sequences had a similarity higher than 80% (Figure 1D). The majority of the annotated sequences corresponded to the known nucleotide sequences of plant species with 83.14%, 6.46%, 2.87%, 0.63%, and 0.36% matches with *Populus trichocarpa*, *Ricinus communis*, *Vitis vinifera*, *Glycine max*, and *Medicago truncatula* respectively.

The size distribution of the BLAST-aligned coding sequence (CDS) and predicted proteins are shown in Figure S2A, B, respectively. The remaining 41.49% (20,256/48,817) of unigenes that did not match sequences in the databases were analyzed by ESTScan to predict coding regions. An additional 12,740 unigenes (62.89%) also showed orientation in the transcriptome coding sequence (Figure S2C, D). The sequences without a homologous hit may represent novel genes specifically expressed in willow stem shoots, or they could be attributed to other technical or biological biases such as assembly parameters. Furthermore, some cDNA are non-coding, lineage-specific or highly variable and need to be further verified.

**Figure 2:**
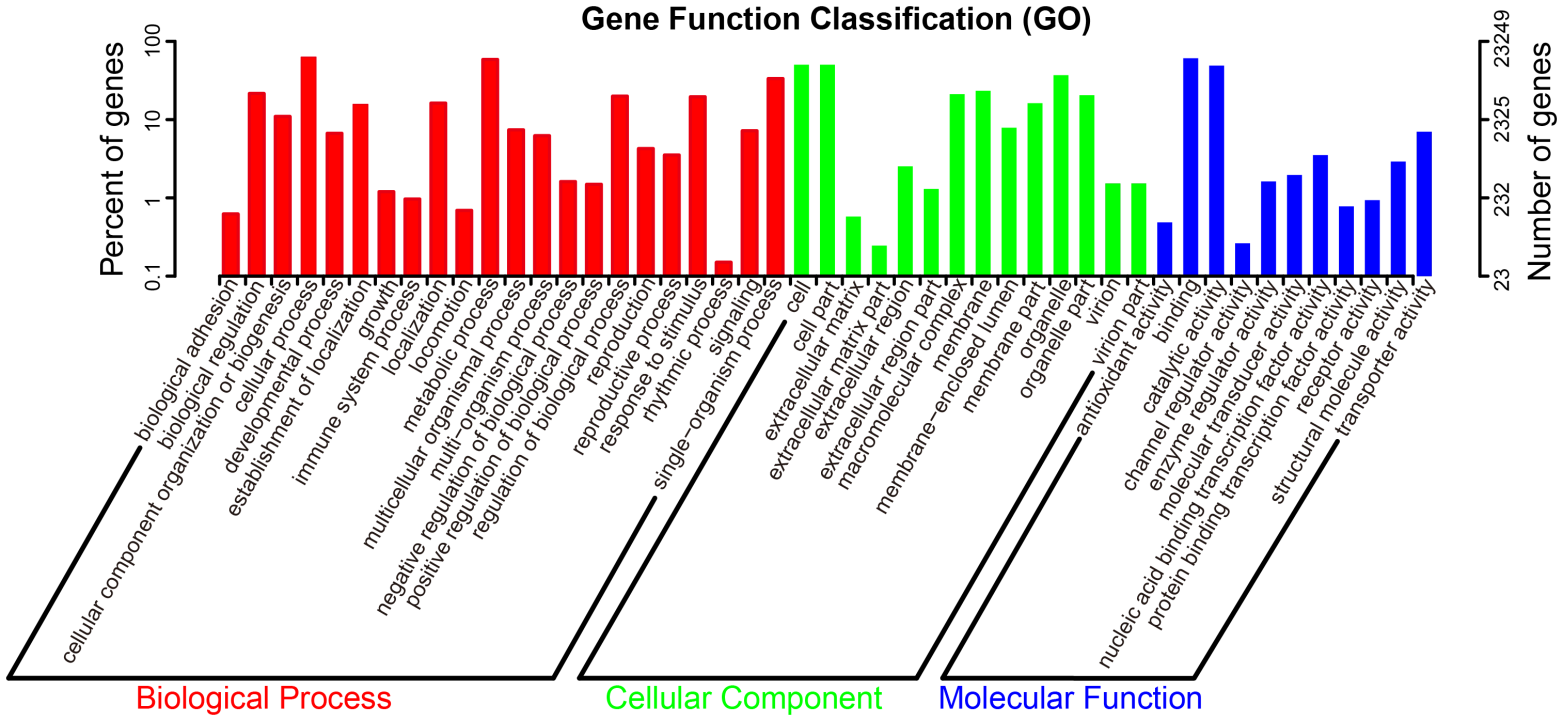
Number of unigenes in each functional category. Unigenes were classified into different functional groups based on a set of plant specific GO terms within biological process, cellular component, and molecular function.

GO annotation is an international classification system that can provide standardized vocabulary for assigning functions of the uncharacterized sequences [16]. A total of 23,249 unigenes (47.62% of all the assembled unigenes) were assigned at least one GO term. GO annotation assignments classified 21,577 unique contigs into 22 subcategories of the biological process category, 11 subcategories of the molecular function category, and 14 subcategories of the cellular component category at level 2 (Figure 2). Among biological processes, transcript sequences assigned to biological regulation, cellular process, metabolic process and single-organism were the most abundant. The subcategories with the most highly abundant transcripts in the cellular components category included cell, cell part, macromolecular complex, membrane, and organelle. Within the molecular function category, the majority of the GO terms were predominantly assigned to the binding and catalytic activity.

**Figure 3:**
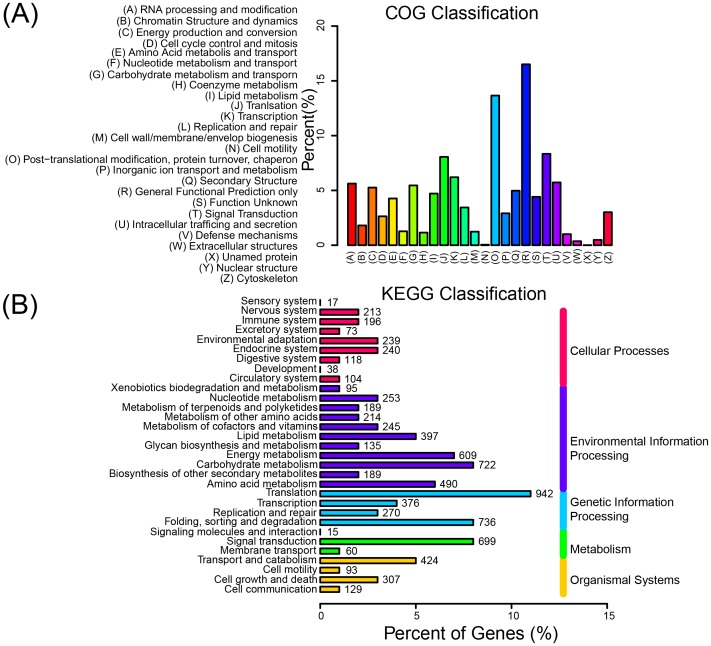
COG and KEGG function classification of unigenes. (**A**) COG function classification of unigenes shows 26 different functional groups. (**B**) Five subcategories (cellular processes, environmental information processes, genetic information processes, metabolism, and organismal systems) were classified by KEGG function classification.

The database of COGs is a phylogenetic classification of protein encoded in completely sequenced genomes [17]. Overall, 10,073 of 48,817 (20.63%) unigenes were assigned to the COG classification (Table 2). Among the 26 COG categories, the cluster for ‘general function prediction only’ (1,663, 14.66%) associated with the basic physiological and metabolic functions represented the largest group, followed by post-translational modification, protein turnover, chaperons (1,376, 12.13%), signal transduction (840, 7.40%), ‘translation (812, 7.15%), and intracellular trafficking and secretion (577, 5.08%). However, only a few unigenes were assigned to cell motility (5, 0.04%), extracellular structures (37, 0.33%), and nuclear structure (49, 0.43%). One unigene was annotated as ‘unnamed protein’ (Figure 3A).

KEGG is a database resource that integrates genomic, chemical and systemic functional information that can facilitate to understand the biological functions of genes in terms of networks [18]. The assembled unigenes were annotated with KEGG Orthology (KO) numbers using BLASTx alignments against KEGG with a cut-off E value of 10^−5^. Overall, 5,444 of 48,817 (11.15%) unigenes were matched in the database and were assigned to 242 KEGG pathways. Among the five categories of pathway hierarchy 1, the ‘metabolism’ represented the largest group, which were sorted into 11 subcategories including carbohydrate metabolism, energy metabolism, amino acid metabolism, lipid metabolism, nucleotide metabolism, and some others (Figure 3B).

### Detection of differentially expressed genes between two transcription libraries

**Figure 4:**
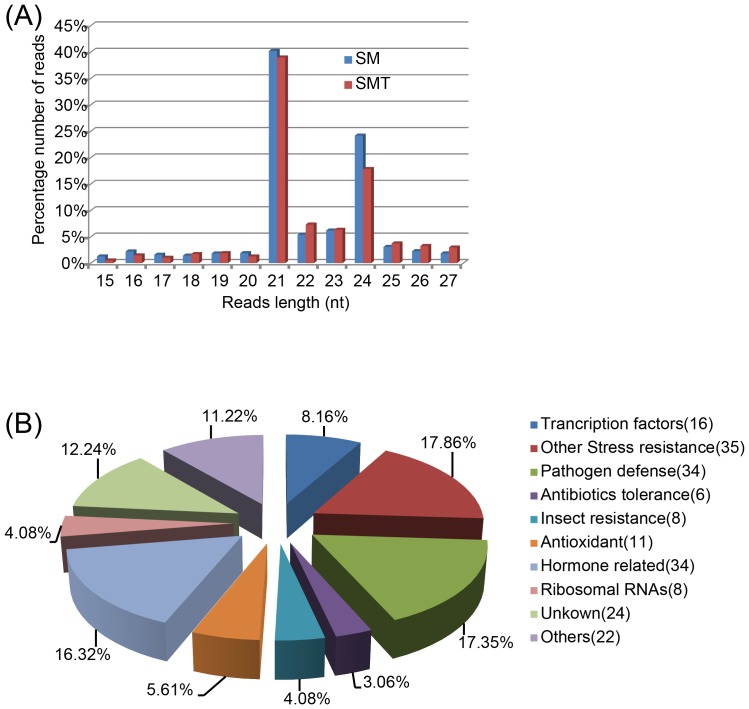
Classification of DEGs and sequence length distribution of small RNA libraries of SM and SMT. (**A**) Up-regulated genes in SM compared to SMT. (**B**) Sequence length distribution shows 21 and 24-nucleotides reads were of significant greater proportion than others.

A quality control test for the data assembled from each cDNA library confirmed that they were suitable for statistical analysis for differentially expressed genes identification (Figure S3). A total of 292 differentially expressed genes were detected between SM and SMT groups when log_2_ (fold change) >1 and P<0.005 were used as cutoff values. Further analysis showed that 196 genes involved in stress resistance, pathogen defense, antibiotics tolerance, insect resistance, antioxidant, hormone related and ribosomal RNAs were up regulated (Figure 4A), whereas 96 genes involved in cell wall synthesis, photosynthesis, stress resistance and antibiotics tolerance were down regulated in SM as compared to SMT. Most of the differentially expressed genes are related to plant adaption stress which includes drought, cold, salt, pathogen, insect, and antibiotics. Among the 196 up regulated genes, 133 genes (67.86%) are related to stress adaption indicating that *S. matsudana* has a better ability to tolerant harsh environment.

### Deep sequencing of small RNA

Two libraries were constructed for small RNA deep sequencing, and we identified 12,532,326 and 20,113,588 sequence reads respectively from SM and SMT. Among the two data sets, the most abundant classes of small RNA found were 21 and 24 nt, which concurs with most reports of deep sequencing of small RNA in plants (Figure 4B) [19]–[21]. The sRNA were classified into eight categories (miRNA, tRNA, rRNA, snRNA, snoRNA, ta-siRNA, repeats, and others) by comparing the acquired to miRBase (http://microrna.sanger.ac.uk/sequences), Rfam (http://rfam.sanger.ac.uk/) and Genbank (http://www.ncbi.nih.gov/Genbank/) (Figure S4). Besides the unknown sRNA, miRNA made up 16.25% and 15.94% of the total acquired sequences respectively, which were represented in the main by 21 nt small RNAs. There was little variation in the different sRNA categories between SM and SMT (SD = 2.31%) indicating that sRNA categories are relatively stable in the two willow species.

### Known miRNA and comparison of miRNA expression levels between the two libraries

A total of 166 known miRNAs belonging to 35 families were obtained in SM and SMT. These miRNA exhibited variable abundance. Deep sequencing analysis is a powerful strategy that allows the identification and quantification of differences in the expression of sRNA. In the 35 families, miR159 was the most accumulated miRNA with a total of 410,956 reads detected between the two libraries (87,687 miRNAs in SM and 323,269 in SMT) (Table 3). There was considerable expression level diversity between the families probably because the willow one-year-old stem is a highly differentiated organ and the genes are in the process of dynamic expression during development. For instance, miR159, miR166, miR319, and miR396 were sequenced more than one hundred thousand times while miRNAs, miR399, miR408, miR481, miR1446, and miR6459 were detected less than 10 times. Members of the same family were also not expressed equally. The two libraries, SM and SMT, had 36 miRNAs that were differentially expressed, among which 24 were up regulated, and 12 were down regulated in SM compared to SMT.

**Table 3:** Conserved miRNA families expression in SM and SMT.

Family	Mature sequence	Total	SM		SMT	
			Raw	TPM	Raw	TPM
miR156	UUGACAGAAGAUAGAGAGCAC	23	5	18	18	42
miR159	UUUGGAUUGAAGGGAGCUCUA	410,956	87,687	323,269	175,659	406,596
miR160	UGCCUGGCUCCCUGUAUGCCA	905	193	712	181	419
miR162	UCGAUAAACCUCUGCAUCCAG	7,920	1,690	6,230	909	2,104
miR164	UGGAGAAGCAGGGCACGUGCA	1,237	264	973	112	259
miR166	UCGGACCAGGCUUCAUUCCCC	140,711	30,024	110,687	28,821	66,712
miR167	UGAAGCUGCCAGCAUGAUCU	1,219	260	959	92	213
miR168	UCGCUUGGUGCAGGUCGGGAA	4,312	920	3,392	374	866
miR171	UGAUUGAGCCGUGCCAAUAUC	366	78	288	37	86
miR172	AGAAUCCUGAUGAUGCUGCAG	984	210	774	127	294
miR319	UUGGACUGAAGGGAGCUCCC	335,098	71,501	263,597	154,043	356,562
miR390	AAGCUCAGGAGGGAUAGCGCC	1,875	400	1,475	201	465
miR393	UCCAAAGGGAUCGCAUUGAUC	347	74	273	15	35
miR394	UUGGCAUUCUGUCCACCUCC	7,217	1,540	5,677	144	333
miR396	UUCCACAGCUUUCUUGAACUU	153,993	32,858	121,135	14,009	32,427
miR398	UUCUCAGGUCACCCCUUUGGG	951	203	748	983	2,275
miR399	UGCCAAAGGAGAUUUGCCCGG	5	1	4	1	2
miR403	UUAGAUUCACGCACAAACUCG	17,261	3,683	13,578	233	539
miR408	AUGCACUGCCUCUUCCCUGGC	5	1	4	0	0
miR472	UUUUCCCUACUCCACCCAUCCC	22,304	4,759	17,545	2,568	5,944
miR475	UUACAAUUCCAUUGAUUAAACCGU	19	4	15	11	25
miR481	AGGACCUCACCUAACAGCUUAAGC	9	2	7	0	0
miR482	UUGCCUACUCCACCCAUGCCAC	731	156	575	102	236
miR530	UGCAUUUGCACCUGCACCUUC	506	108	398	53	123
miR828	UCUUGCUCAAAUGAGUAUUCCA	37	8	29	5	12
miR1444	UCCACAUUCGGUCAAUGUUC	42	9	33	3	7
miR1446	UUCUGAACUCUCUCCCUCAA	5	1	4	0	0
miR2111	UAAUCUGCAUCCUGAGGUUUG	23	5	18	4	9
miR6421	UAGAGCAGAUUGUAAGGGAAG	28	6	22	24	56
miR6427	UCGUAAUGCUUCAUUCUCACAA	173	37	136	40	93
miR6445	UUCAUUCCUCUUCCUAAAAUGG	8,586	1,832	6,754	1,215	2,812
miR6457	UAAUCUCUCUGCAGAAUGCUG	0	0	0	28	65
miR6459	UCGAAUUUGGGCUUGAGAUUG	9	2	7	2	5
miR6474	UGUUCAGAUCAGUAGAUAGCA	80	17	63	21	49
miR6476	UCAGUGGAGAUGAAACAUGA	0	0	0	1	2

### Identification of new miRNAs

Plant miRNA research has significantly progressed because of deep sequencing technology; however, stricter requirements are needed for identifying a miRNA candidate as a reliable new miRNA. Altogether, we obtained 31 predicted novel miRNAs by using two additional criteria [22]. Among these novel miRNAs, 28 and 30 were expressed in SM and SMT, respectively. The majority of the miRNAs (18 of the 31, 58.06%) have a uridine at 5′ terminal, showing a preference of AGO1 [23]. The novel miRNAs also displayed unequal expression levels as known miRNAs between the two libraries (Table S1). As none of the novel miRNAs had homologs in miRBase, we presumed that they were *S. matsudana*-specific, or were restricted to closely related species.

### miRNA Targets predicting and sequences validating

**Figure 5:**
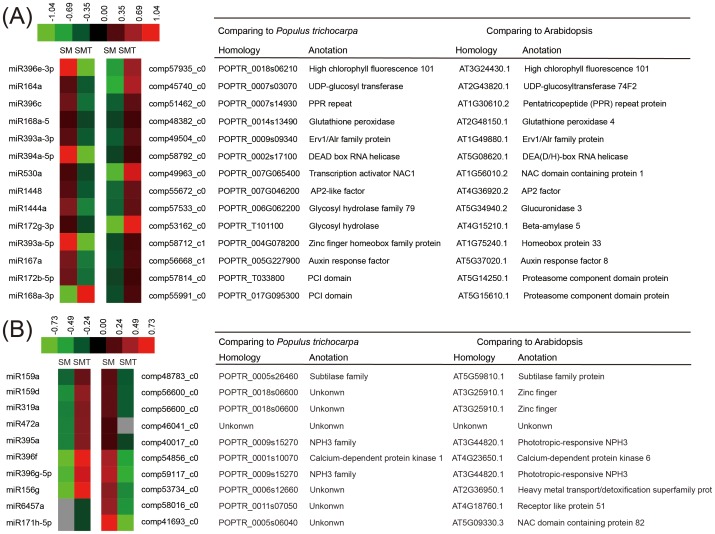
A combined view of reverse expressions between miRNA and its target. The left side of hotspot figure show miRNA expression level, and the right side show corresponding target expression levels of both SM and SMT. Up and down regulation in the expression were based on normalize data (color bar at the top) generated by Cluster 3.0 software. The tables show homologous genes of miRNA target genes by comparing to *Populus trichocarpa* and Arabidopsis. (**A**) Reverse expression of Up-regulated miRNA in SM and its targets. (**B**) Reverse expression of down-regulated miRNA in SM and its targets.

Transcriptome of SM and SMT was used to identify miRNA targets. The 35 known miRNA families had 108 affiliated target genes while the 31 novel miRNAs did not target any genes. Functional annotation of the targets was found to be diverse and included the response to stimulus, signal transduction factors, transcription factors, and genes involved in other biological processes. Among the different expressed targets, 24 miRNAs-target pairs showed reverse expression changing pattern when the results from miRNA profiling and the transcriptome profiling were compared (Figure 5). Functional annotations showed that the 24 miRNA-targets pairs were involved in auxin signaling, stress tolerance, signal transduction factors, and other proteins were involved in various biological processes.

**Figure 6:**
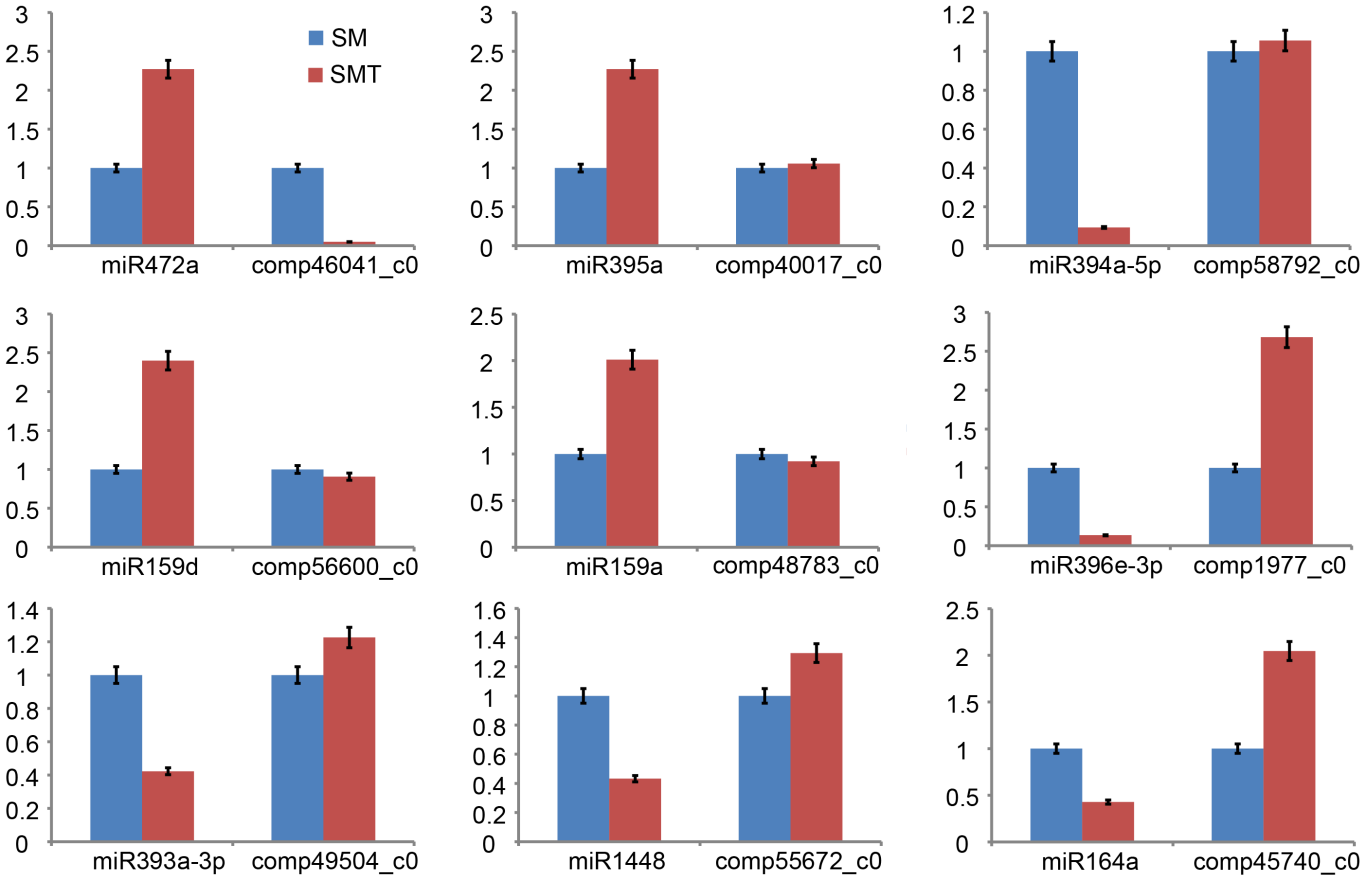
Real-time quantitative PCR validate expressions of 9 selected miRNA and its targets. The amount of miRNA expression was normalized to the level of 5.8rRNA, and the mRNA expression was normalized to the level of *Populus* actin gene.The normalized miRNA and its target gene levels of SM were arbitrarily set to1.

To validate the reliability of RNA-seq, full-length cDNA sequences of twenty selected genes were isolated by T-A cloning and compared with the assembled sequences. The length of these genes ranged from 192 to 1,960 bp, and the sequence variation was minimal. All of the genes had the sequence pairwise identity bigger than 98% (Table S2), which validated the reliability of RNA-seq. To partially validate the miRNA and its target genes expression levels, nine miRNA-target pairs were randomly selected for a real-time qPCR experiment. The results showed that the expression of miRNA target pairs changed trends as compared with the sequencing results (Figure 6).

## Discussion

### Overview of the deep sequencing datasets

Although many studies have reported the transcriptome analysis of willow, here have been no studies on mRNA and miRNA expression profiles in *S. matsudana.* In this study, the RNA-seq technology was used to generate transcriptome and small RNAome data and examined the global gene expression profiling of shoot tips from *S. matsudana*. This work demonstrates that RNA-seq is a useful and effective tool for *de novo* transcriptome and small RNA assembly. Moreover, it facilitates the identification of differentially expressed miRNAs and their target genes between the two willow cultivars, even in the absence of a genomic database. We generated both mRNA and siRNA libraries from the shoot tips of the two willow species to substantially increase the available data on willow mRNAs and sRNAs to obtain genomic resources for further investigating candidate genes especially the stress resistance genes in various metabolic pathways in willow species. Comparison of transcriptome sequence data of the two libraries revealed 292 differentially expressed genes with over 67% involved in stress response. Differential expression of miRNAs also showed many miRNAs and their target genes were stress related. The real-time PCR analysis of the differentially expressed miRNAs and genes further verified the miRNA and the transcript expression level revealed by small RNA and transcriptome comparison from RNA-seq data.

### Transcription factors related to stress tolerance in *S. matsudana*

Transcription factors play a key role in modulating the acclimation response of plants to various internal or external cues. They potentially control downstream gene expression in stress signal transduction pathways through activation and repression of the genes after certain stress treatments. Plant genomes contain a large number of transcription factors (TFs). It has been estimated that the Arabidopsis genome codes for at least 1,533 transcription factors and over 5.9% of its total estimated genes. Among these, about 800 genes encode AP2/EREBP, bZIP, zinc finger proteins, Myb proteins, and AUX/IAA types of transcription factors. Sixteen transcription factors have higher transcription levels in SM compared to SMT, which include C-repeat binding factors (CBF), Myb proteins, TINY proteins, AP2/EREBP, WRKY type, and other types of zinc finger proteins.

CBF proteins bind to the cold-responsive *cis*-acting elements that contain the same motif (CCGAC), are encoded by AP2/EREBP multigene families, and mediate the transcription of genes in response to stress. Overexpression of two Arabidopsis AP2/EREBP genes, CBF1 and DREB1A, resulted in stronger tolerance to drought, salt, and freezing [24]–[26]. Transgenic Arabidopsis plants overexpressing CBF3 under the control of cauliflower mosaic virus (CaMV) 35S promoter also showed a high tolerance to drought, high-salinity, and freezing stress [25], [27], [28]. Five CBF and AP2/EREBP genes were highly expressed in SM as compared to SMT. The sequenced CBF and AP2/EREBP gene reads in SM are 2 to 30 times over that in SMT indicating that the SM may have a high tolerance to abiotic stress.

WRKY family is defined by a DNA binding domain that contains the strictly conserved amino acid sequence WRKY. Members of WRKY family transcription factors have been implicated in the response to biotic and abiotic stresses. Overexpression of the GhWRKY39-1 gene enhances the resistance to pathogen infection and tolerance to high salt and oxidative stress in transgenic Nicotiana benthamiana [29]. The same results were obtained from the research of *Tamarix hispida*, overexpression of ThWRKY4 conferred tolerance to salt, oxidative and ABA treatment in transgenic plants [30]. The overexpression of a subgroup IIe WRKY transcription factor CaWRKY27 of *Capsicum annuum* also positively regulates the resistance of tobacco to the pathogen *Ralstonia solanacearum* infection [31]. In the present study of the two transcriptome libraries, three WRKY showed higher expression levels in SM as compared to SMT that may enhance the tolerance of SM to biotic and abiotic stress.

Plant zinc finger transcription factors play an important role in tolerance to various environmental stresses such as drought, cold, osmotic stress, wounding and mechanical loading. The zinc finger proteins chraracterized by zinc finger domains enable protein interaction with DNA. A large number of studies have shown that zinc finger proteins may not be specific to one particular stress, but may regulate responses to several stresses. In *A. thaliana*, the zinc finger proteins have been shown to be involved in cold, dehydration, reactive oxygen, and salt stress [32], [33]. In *Populus euphratica*, the overexpression of a zinc finger protein gene *PSTZ* showed enhanced salt tolerance [34]. In rice, seven zinc finger transcription factors were involved in the response to different abiotic stresses such as cold, drought, and mechanical stress [35]. In *Capsicum annuum*, a zinc finger transcription factor gene functions as a pathogen induced early-defense gene [36]. Six zinc finger transcription factors have a higher transcription level in SM than SMT indicating that the SM has a stronger ability to tolerate abiotic stresses.

### Hormones related genes involved in stress tolerance in *S. matsudana*

Plants hormones such as auxins, abscisic acid (ABA), gibberellins (GA), salicylic acid (SA), ethylene (ET), cytokinins (CK), jasmonates (JA), and brassinosteroids (BR) play crucial roles in various biotic and abiotic responses. A vast array of pathogenic microorganisms which deliver virulence factors into plant cell to promote virulence and cause disease and plant can change the level of various phytohormones to protect the cell from infection when plants were attacked by these diverse pathogens [37], [38]. Plants hormones also have the ability to adapt changing environments by mediating a wide range of adaptive responses [39], [40]. It is notable that xx hormone-related transcripts have significantly higher expression levels in SM than in SMT, which include eighteen ABA related genes, ten auxin response genes, four JA, and two ET related genes.

Under abiotic stresses such as drought, cold, and salinity, ABA levels increased in vegetative tissues, which are adaptive responses essential for the plant survival and productivity [41]. In Arabidopsis, increasing the transcription of a key ABA biosynthesis gene, *NCED3*, resulted in the increased production of ABA and enhanced tolerance to drought-shock and osmotic stress [42]. Overexpression of *AtTRE1* gene that encodes the Arabidopsis trehalase conferred the ability to tolerate drought stress through increased sensitivity toward ABA-dependent stomatal closure [43]. A maize calcium-dependent protein kinase gene, *ZmCPK4*, positively regulates ABA signaling and enhanced drought stress tolerance in transgenic Arabidopsis [44]. Apart from the vital function for abiotic stress tolerance, ABA plays a modulating role in plant pathogen infections [45]–[47]. Many key components of ABA signal transduction have been identified including protein kinases, RNA processing factors, phosphatases, proteasome components, and chromatin remodeling proteins [48], [49]. In *S. matsudana*, thirteen genes involved in the response to ABA transduction had a higher transcription level than SMT. Moreover, five miRNAs, which target five ABA transduction genes were down regulated as compared to SMT. These genes could increase the ABA levels and enhance the ability of *S. matsudana* to tolerate abiotic and biotic stresses.

Auxin responsive genes also regulate plant stress response. Overexpression of an auxin responsive gene *GH3-8* resulted in stronger pathogen resistance in rice and this resistance was shown to be independent of SA and JA signaling [50]. A rice auxin responsive gene *OsGH3.13*, encoding IAA-amido synthetase was shown to enhance the expression of genes that confer drought tolerance [51]. Ten genes related to auxin response had higher transcription levels in SM than SMT, and one miRNA had a lower expression level in SM than SMT. These genes could change the auxin levels and confer increased stress tolerance ability upon *S. matsudana.* Auxin plays a major role of in integrating the activities of multiple phytohormones to control plant growth in response to environmental signals [52]. Except ABA and auxin, JA and ET related genes were also detected that had different transcription in SM and SMT, which in turn could result in variable stress response activity. Four JA related genes and two ET related genes had higher transcript levels in SM than SMT, which could enhance the ability of *S. matsudana* to tolerance stress.

### Antioxidants and detoxification genes related to stress tolerance in *S. matsudana*

Biotic and abiotic stresses such as pathogen infection, salt, drought, oxidation and heat stress are accompanied by the formation of reactive oxygen species (ROS). The ROS such as H_2_O_2_, O_2_, and OH^-^ are the main damages of membranes and macromolecules [53]. Plants have developed many antioxidation strategies to scavenge ROS. Enzymes such as thioredoxin, ascorbate peroxidase (APX), superoxide dismutase (SOD), glutathione peroxidase, glutathione transferases, phospholipid-hydroperoxide glutathione peroxidase, galactose oxidase, and glutathione reductase form a part of the main ROS scavenging strategy. The increased transcript levels of these enzyme genes can result in enhanced tolerance to oxidative stress. In potato, overexpression an APX gene resulted in enhanced tolerance to methyl viologen-induced oxidative stress and high temperature in transgenic potato plants [54]. Overexpression of a tobacco glutathione S-transferase with glutathione peroxidase activity in transgenic tobacco enhanced seedling growth under a variety of stress conditions [55]. In *Solanum tuberosum*, substantial accumulation of a plant thioredoxin named CDSP 32 mRNA and protein were revealed upon oxidative treatments [56]. In *S. matsudana*, eleven genes involved in response to oxidative stress had higher transcription levels than SMT. These genes encode the abovementioned enzymes including one APX, three glutathione transferases, four thioredoxin, one galactose oxidase, and two NADP reductase. The higher transcription levels of these oxidative stress response genes in SM compared to SMT suggestion that SM has stronger stress tolerance.

## Supporting Information

Figure S1 Classification of raw reads of SM and SMT trianscriptiom sequences.
(TIF)

Figure S2 The length distribution of the coding sequence (CDS) and predicted proteins. (A) Aligned CDS by BLASTX. (B) predicted proteins by BLASTX. (C) Aligned CDS by ESTScan. (D) predicted proteins by ESTScan.
(TIF)

Figure S3 Saturation test and RPKM distribution of transcriptome sequences.
(TIF)

Figure S4 Summary of sequence classifications of sequenced sRNA.
(TIF)

Table S1 Mature sequences and expression of novel miRNA.
(XLSX)

Table S2 Primers, sequence length and sequence identity of unigenes validated by real-time PCR.
(XLSX)
