# Supplementary figures and images for: De Novo Transcriptome and Small RNA Analysis of Two Chinese Willow Cultivars Reveals Stress Response Genes in Salix matsudana
Source: PLoS One. 2014 Oct 2;9(10):e109122. doi: 10.1371/journal.pone.0109122 (PMC4183547; doi:10.1371/journal.pone.0109122)

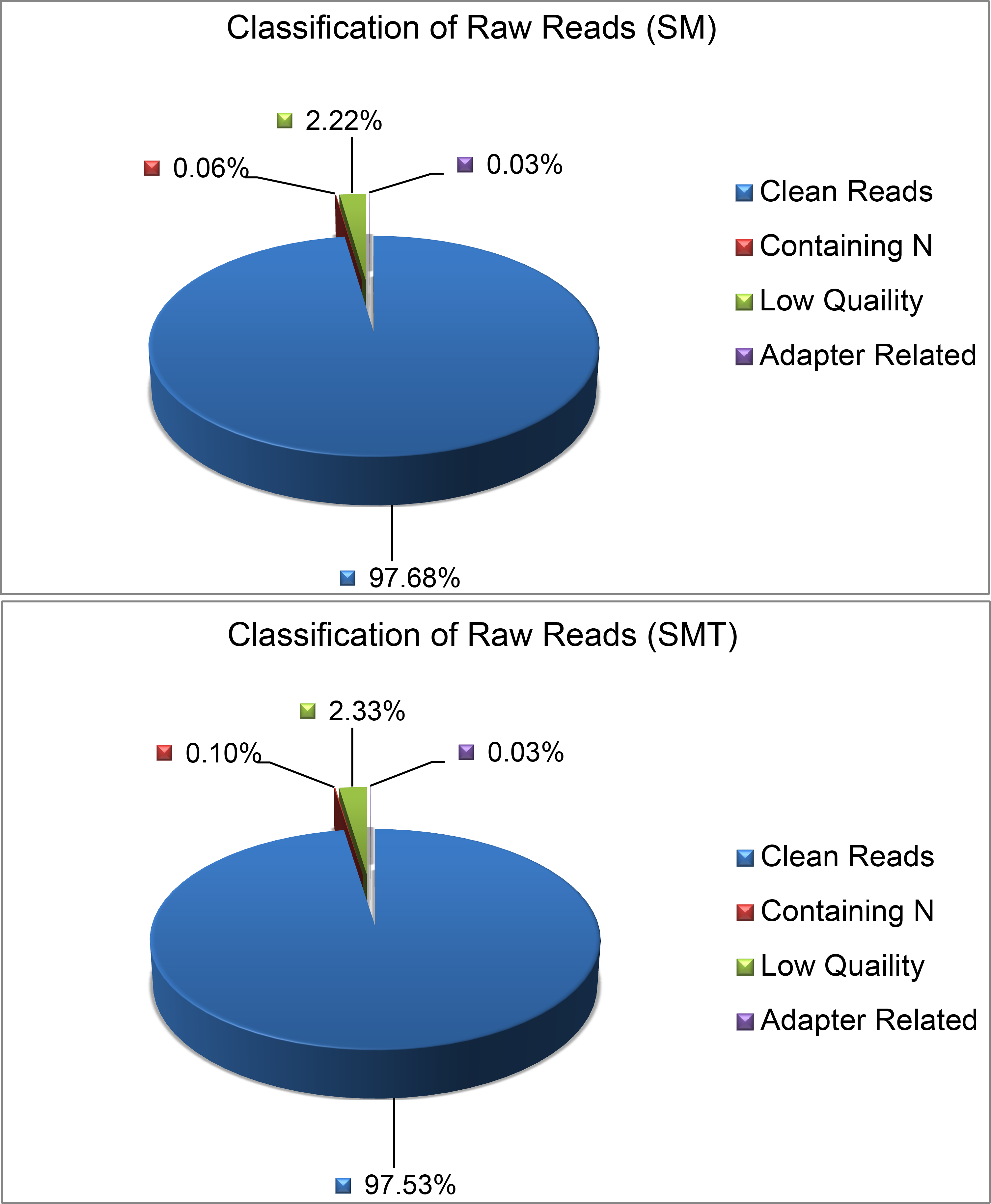

Supplement: Figure S1 — Classification of raw reads of SM and SMT trianscriptiom sequences. (TIF) [file pone.0109122.s001.tif]

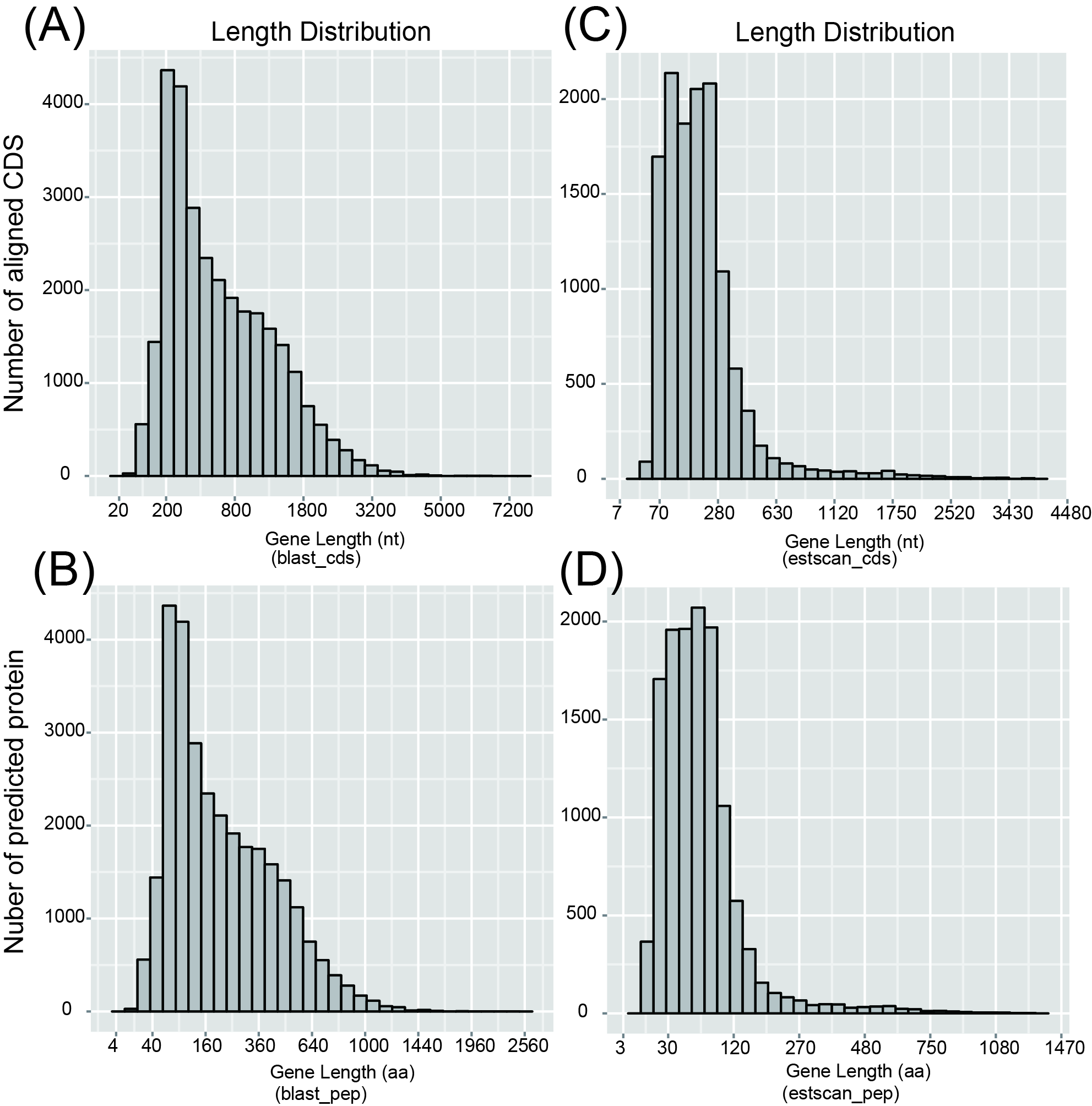

Supplement: Figure S2 — The length distribution of the coding sequence (CDS) and predicted proteins. (A) Aligned CDS by BLASTX. (B) predicted proteins by BLASTX. (C) Aligned CDS by ESTScan. (D) predicted proteins by ESTScan. (TIF) [file pone.0109122.s002.tif]

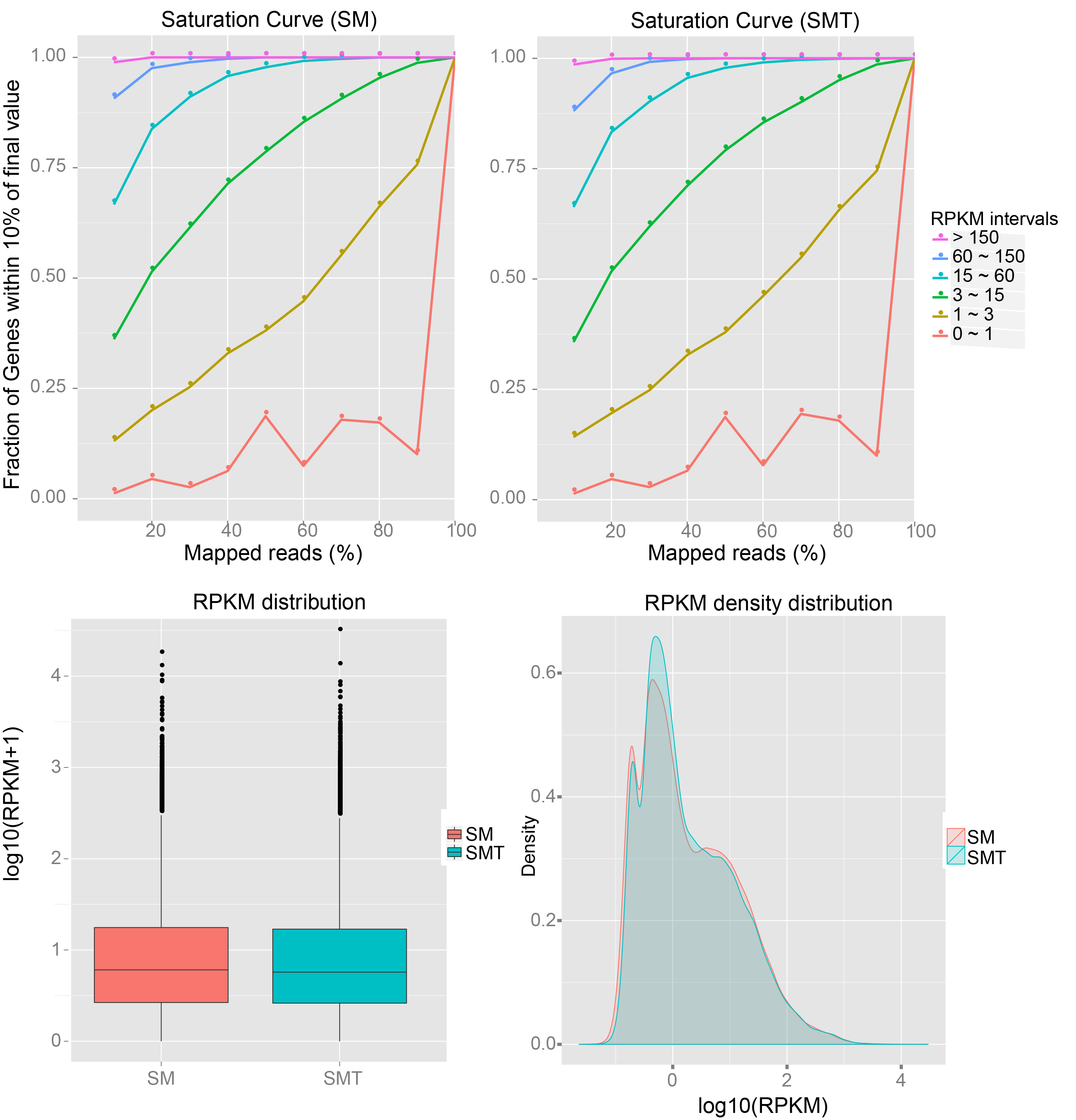

Supplement: Figure S3 — Saturation test and RPKM distribution of transcriptome sequences. (TIF) [file pone.0109122.s003.tif]

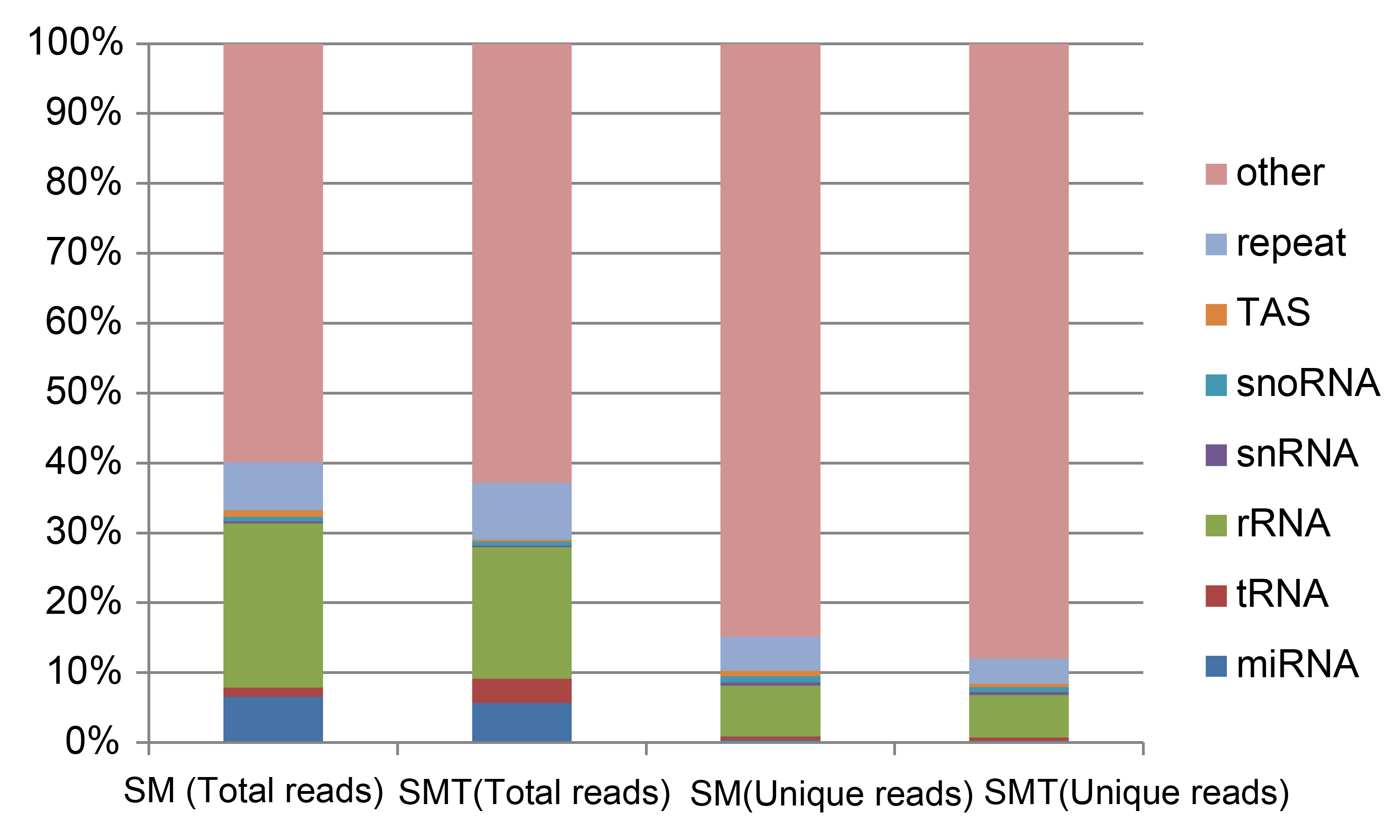

Supplement: Figure S4 — Summary of sequence classifications of sequenced sRNA. (TIF) [file pone.0109122.s004.tif]
